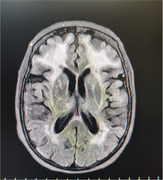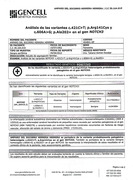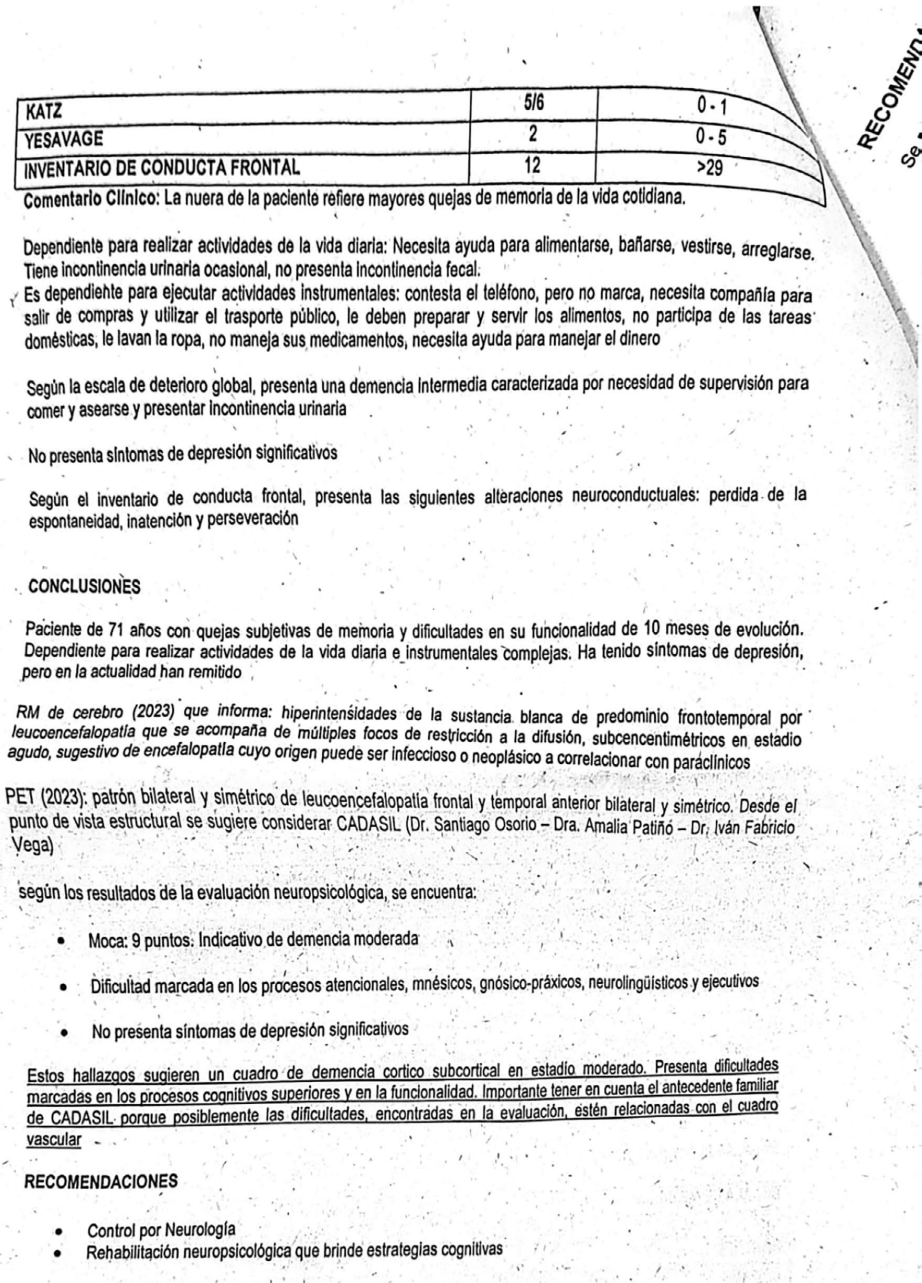# Diagnostic challengein late onset CADASIL with atypical presentation‐case report

**DOI:** 10.1002/alz.086322

**Published:** 2025-01-09

**Authors:** Aury Stella Carrasquilla Romero, Sergio Andres Taborda Holguin

**Affiliations:** ^1^ Universidad del Sinu, Cartagena, Bolivar Colombia; ^2^ Instituto Neurológico de Colombia, Medellin, Antioquia Colombia

## Abstract

**Background:**

This study examines an exceptional case of CADASIL (Cerebral Autosomal Dominant Arteriopathy with Subcortical Infarcts and Leukoencephalopathy), a hereditary cerebrovascular disease caused by a mutation in the notch3 gene. In contrast to typical cases manifesting before the age of 50 with migraines, this report highlights an atypical presentation in a 70‐year‐old woman with no history of migraines nor cognitive impairment.

**Method:**

The patient, with a history of type 2 diabetes, hypothyroidism, and dyslipidemia, was initially treated for cognitive impairment and behavioral changes under suspicion of autoimmune encephalitis. During outpatient follow‐up, the notch3 mutation was documented (Variant c.421c>T; p.Arg141Cys heterozygous NOTCH3 probably pathogenic Code 404841 GP) and confirmed through brain magnetic resonance imaging (MRI) consistent with CADASIL. The clinical examination included neuropsychological assessments and detailed genetic testing.

**Result:**

The patient experienced rapid cognitive deterioration and behavioral changes, with temporary improvements following immunosuppressive treatments. However, complications arose, including pulmonary thromboembolism. Brain MRI revealed hyperintensity in the white matter of frontal and temporal lobes. Genetic analysis identified a probably pathogenic heterozygous variant in the notch3 gene

**Conclusion:**

This case underscores the importance of considering CADASIL in atypical presentations, especially in older patients. Confirmation of the diagnosis through MRI and genetic analysis emphasizes the significance of these diagnostic tools. While proven therapies for improving the disease are lacking, the study highlights the relevance of comprehensive care and specialized follow‐up in CADASIL cases. It concludes with the need for further research in older adult populations to better understand the prevalence and evolution of the pathology in this demographic.